# Patient Preferences for Outcomes Associated With Labor Epidural Analgesia

**DOI:** 10.7759/cureus.22599

**Published:** 2022-02-25

**Authors:** Alison Harding, Ronald B George, Allana Munro, Jillian Coolen, Erna Snelgrove-Clarke, Brendan Carvalho

**Affiliations:** 1 Obstetrics and Gynaecology, Dalhousie University, Halifax, CAN; 2 Department of Anesthesia and Perioperative Care, University of California San Francisco (UCSF), San Francisco, USA; 3 Anesthesiology, Dalhousie University, Halifax, CAN; 4 Department of Women's & Obstetric Anesthesia, IWK Health Centre, Halifax, CAN; 5 Department of Obstetrics and Gynaecology, IWK Health Centre, Halifax, CAN; 6 School of Nursing, Faculty of Health Sciences, Queen’s University, Kingston, CAN; 7 Department of Anesthesia, Stanford University, Stanford, USA

**Keywords:** labor epidural analgesia, pain control, questionnaire study, obstetrical anesthesia, patient preferences

## Abstract

Purpose

Patient preferences for labor epidural analgesia (LEA) have been incompletely evaluated. This study aimed to determine the importance of various LEA outcomes to both antenatal and postpartum patients.

Methods

This was a cross-sectional study approved by the institutional ethics board. Questionnaires were distributed to two separate and distinct cohorts screened for eligibility: pregnant patients at an antenatal visit and postpartum patients during childbirth admission. A list of common LEA outcomes was compiled using research published in leading anesthesia journals. Participants ranked the outcomes according to perceived importance. They assigned each a number from 1 to 10 (priority ranking; 1 indicated the highest priority outcome and 10 the least). They were also asked to ‘spend’ $100 towards the outcomes (relative value scale), allocating more money to outcomes more important to them.

Results

Two hundred twenty questionnaires were completed (105 antenatal, 115 postpartum). ‘Achieving desired pain relief’ was the most important outcome for both cohorts. It was valued more by the postpartum cohort (Median $50 (25 - 60) vs $30 (18 - 50)). ‘Overall satisfaction with the pain management,’ ‘experiencing a short time to achieve pain relief,’ and ‘experiencing a short duration of labor’ received more money than avoiding various LEA-related side effects. The postpartum cohort ranked ‘experiencing a short time to achieve pain relief’ as more important than the antenatal cohort (Median 5 (3 - 7) vs 3 (2 - 5)).

Conclusions

Achieving the desired pain relief was the highest LEA outcome preference for both antenatal and postpartum patients. Avoiding side effects was less important relative to pain-related outcomes.

## Introduction

For many patients, labor analgesia is an important part of the childbirth experience. While there are several modalities to choose from, labor epidural analgesia (LEA) is most often considered the gold standard [1] and is currently utilized by the majority of laboring patients in the United States and Canada [2]. Many studies have confirmed the effectiveness of LEA in relieving the pain of childbirth, however, there is limited literature examining patient preferences with LEA. The majority of studies investigated factors associated with choosing LEA (e.g. previous epidural, fear of childbirth) [3-5] and overall satisfaction with the procedure [6].

In 2005, Carvalho et al. used a questionnaire to investigate the preferences for patients undergoing elective cesarean delivery to determine the importance of several intraoperative and postoperative anesthesia outcomes [7]. However, patients’ specific preferences for LEA have not yet been effectively evaluated. Involving patients in clinical and research partnerships ensures their ability to influence decisions based on their priorities and builds a culture of engagement, however, this is currently under-utilized in obstetric anesthesia care [8]. Informing healthcare providers of patient preferences may help them to better understand their patients’ desires and guide the care they provide to meet the patients’ expectations.

The primary objective of this study was to determine patient preferences for maternal outcomes associated with the use of LEA. Secondarily, we evaluated patients’ expectations from or satisfaction with their LEA experience and its relation to their preferences.

This article was previously presented as a poster presentation at the Dalhousie University Anesthesia Research Day (April 2019), the Society for Obstetric Anesthesia & Perinatology Annual Meeting (May 2019), the Canadian Anesthesiologist Society Annual Meeting (June 2019), and the Society of Obstetricians and Gynaecologists of Canada Annual Clinical and Scientific Conference Medical Student Program (June 2019).

## Materials and methods

Institutional research ethics board approval was obtained on July 3, 2018 (IWK REB #1023225). The institutional research ethics board waived the need for written informed consent, as the completion of the questionnaire was considered consent to participate. The study was conducted at the IWK Health Centre in Halifax, NS, Canada. The IWK Health Centre is a women’s and children’s hospital with approximately 4,800 deliveries per year and an LEA rate of 60-80%.

Questionnaire development

For this cross-sectional study, we created a novel questionnaire as the primary research tool. We first developed a list of relevant maternal outcomes related to LEA. The initial list was created by a targeted literature search of the common outcomes measured in randomized controlled trials of LEA published over a two-year period from leading anesthesia and obstetric journals (Anesthesia & Analgesia, Anesthesiology, International Journal of Obstetric Anesthesia, Obstetrics and Gynecology, Canadian Journal of Anesthesia, American Journal of Obstetrics and Gynecology, and British Journal of Anaesthesia). The table of contents of each issue from the above journals published between June 2016 and May 2018 was searched for randomized controlled trials containing the terms “epidural”, “labour”, or “labor” in the title. The abstract of each study identified was examined to determine relevance; if relevant, the outcomes used by the study were recorded. After examining all issues from the five journals in the specified timeframe, the number of times each outcome was used was totaled. Ten of these outcomes were selected by the research team for the questionnaire based on the frequency with which they appeared in the previous literature and expert opinion as to which would be most relevant. We chose 10 outcomes, as this is consistent with previous literature [7] and felt to be manageable for participants in a 10-minute questionnaire. The 10 outcomes used in the questionnaire, with the explanations written in lay terms by the research team, are presented in Table 1.

**Table 1:** Labor epidural analgesia (LEA) evaluated outcomes as presented and defined in the study questionnaire

Description of Possible Outcome
Achieving desired pain relief: You receive the amount of pain relief you want from the labor epidural.
Avoiding complications such as low blood pressure: You do not experience any dizziness or light-headedness during labor.
Avoiding itching as a side effect: You do not experience any itching that is not relieved by scratching during labor.
Avoiding nausea and/or vomiting as a side effect: You do not experience the feeling of being sick to your stomach during labor.
Receiving the smallest effective dose of pain medication: The amount of medication you receive is not more than what is needed.
Overall satisfaction with the pain management: You are generally happy with the labor epidural experience.
Experiencing a short duration of labor: The time you spend laboring is no longer than you expect.
Avoiding leg weakness as a side effect: You do not experience excessive leg heaviness that affects your ability to walk and turn in the bed during labor.
Experiencing a short time to achieve pain relief: The time between the placement of the epidural and desired pain relief is no longer than you expected.
Avoiding anxiety related to labor pain: You do not experience any increased worry or fear due to pain during labor.

The LEA outcomes were evaluated for importance by participants using methods similar to those used in the questionnaire published by Carvalho et al. [7], which evaluated patient preferences prior to cesarean delivery. This technique was chosen to maintain consistency between publications. Participants were first asked to rank the outcomes according to their perceived importance to them as individuals, assigning each a number from 1 to 10 (priority ranking), where 1 indicated the highest priority outcome and 10, the lowest priority. They were required to use all 10 numbers, with no repetition. Participants were also asked to ‘spend’ a theoretical $100 toward the outcomes (relative value scale) [9], allocating more money to outcomes more important to them and less to outcomes less important to them. They were allowed to spend any amount on any outcome, including $0, as long as the total allocated to all 10 outcomes equaled $100.

Investigators collected participant demographics, including age, household income, education level, and marital status, in addition to obstetric history and past experience with childbirth pain management. The questionnaires were distributed to two separate cohorts, an antenatal group and a postpartum group. The postpartum questionnaire differed slightly, as it included questions regarding the participants’ recent labor experience.

The questionnaire also assessed LEA expectations (antenatal cohort) or satisfaction with the LEA experience (postpartum cohort) using three 1 - 10 numerical ranking scale questions. These questions asked about expected or experienced satisfaction with the procedure, expected or experienced pre-LEA pain level, and expected or experienced post-LEA pain level.

Prior to implementation, the questionnaire was informally tested on two anesthesiologists, one obstetrician, and two patients to confirm readability, content, and face validity. The questionnaire was developed to take approximately 10 minutes to complete.

Data collection

Data were collected from July to October 2018 at the IWK Health Centre in Halifax, NS. Potential participants for the antenatal cohort were identified prior to an appointment at the IWK Health Centre’s Prenatal Clinic via a chart evaluation conducted by the research team. Patients eligible for participation were required to be English speaking, non-laboring, carrying a singleton pregnancy greater than 24 weeks gestation, an American Society of Anesthesiologists (ASA) Physical Status Classification < 4, expecting a term vaginal birth, and open to the possibility of an LEA (antenatal cohort) or recently used LEA (postpartum cohort). If eligible, a study reminder sheet was placed in the patient’s chart and during their antenatal appointment, a member of their care team introduced the study and invited them to participate. If interested in participating, a member of the research team would then meet with them after their antenatal appointment to discuss the study and administer the questionnaire.

The postpartum cohort was introduced to the study by their anesthesiologist and asked if a member of the research team may contact them the day after their delivery. Patients were excluded if there were known fetal abnormalities, they experienced complications of delivery resulting in harm to the parturient or intensive care admission for the neonate, or previously participated in the study’s antenatal cohort. A member of the research team would meet the patient one day after delivery to discuss the study and administer the questionnaire.

The questionnaires were completed by participants with pen and paper, and a member of the research team was available as needed to answer questions while the participant completed the questionnaire. The research team met with each participant only once, no contact information was collected and there was no scheduled follow-up with participants.

Statistical analysis

As this study is descriptive and exploratory in nature, a target sample size of 100 participants in each cohort, for a total of 200 participants, was selected based on feasibility. We estimated that this number would be obtained in a four-month period. Descriptive statistics were used to summarize the demographic data, the frequency at which each outcome was each given rank from 1 to 10, the median amount of money each outcome was assigned, and the median scores assigned in the satisfaction section. The data were tested for normal distribution using the Shapiro-Wilk test. As more than half of our data did not meet the assumption of normality, all data are presented as median (interquartile range).

## Results

**Figure 1:**
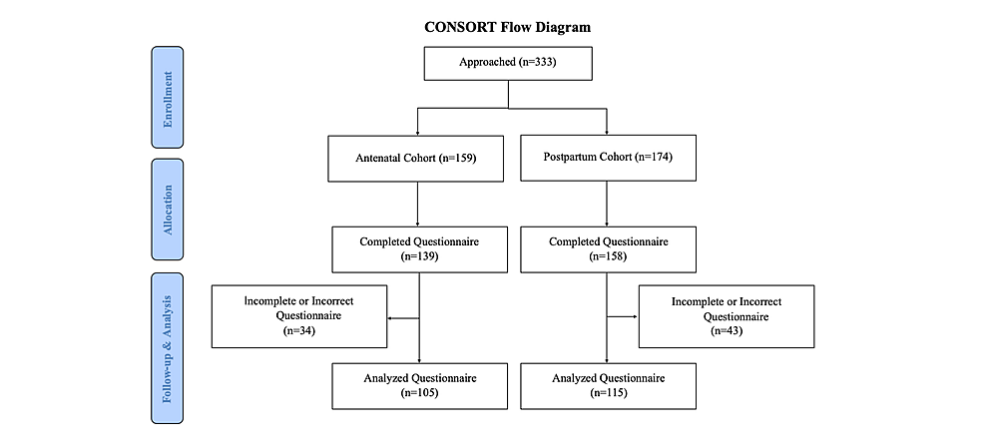
CONSORT patient flow diagram

We approached 333 patients for participation in the study period from July to October 2018, 159 patients in the antenatal cohort, and 174 in the postpartum cohort. Of these, 297 completed the questionnaire, 139 in the antenatal cohort, and 158 in the postpartum cohort (89% response rate overall). Seventy-seven questionnaires were not completed correctly due to errors with allocating outcome priority ranking and relative value scores. The remaining 220 questionnaires were completed correctly by participants, 105 in the antenatal cohort and 115 in the postpartum cohort (Figure 1).

Demographics of the 220 study participants in each distinct cohort are presented in Table 2. The majority of the study population was Caucasian, married or living with their partner, and highly educated, having completed some form of secondary education. Over half the participants in both cohorts reported a household income greater than $88,000 per year, and most of the participants were nulliparous or just delivered their first baby.

**Table 2:** Demographical data of study participants * N = 115 for the postpartum cohort. † N = 105 for antenatal cohort. N = number, % = percentage, NA = not applicable.

		Antenatal (N = 105)	Postpartum (N = 115)
Age (Mean ± standard deviation)		32 ± 5	30 ± 5
Ethnicity			
	Caucasian (n (%))	77 (73.3)	99 (86.1)
	Non-Caucasian (n (%))	28 (26.7)	16 (13.9)
Education ^*^			
	Did not complete high school (n (%))	3 (2.9)	3 (2.6)
	High school diploma (n (%))	4 (3.8)	14 (12.3)
	Community college degree (n (%))	21 (20.0)	29 (25.4)
	University degree (n (%))	47 (44.8)	44 (38.6)
	Master/PhD (n (%))	17 (16.2)	12 (10.5)
	Second University Degree (n (%))	9 (8.6)	9 (7.9)
	Other (n (%))	4 (3.8)	3 (2.6)
Relationship Status			
	Married (n (%))	78 (74.3)	68 (59.1)
	Living with partner (n (%))	22 (21.0)	38 (33.0)
	Other (n (%))	5 (4.9)	9 (7.8)
Income			
	< $ 39 000 (n (%))	8 (7.6)	14 (12.2)
	$ 39 000 – 62 000 (n (%))	11 (10.5)	20 (17.4)
	$ 62 000 – 88 000 (n (%))	12 (11.4)	11 (9.6)
	$ 88 000 – 125 000 (n (%))	33 (31.4)	31 (27.0)
	> $ 125 000 (n (%))	36 (34.3)	28 (24.3)
	Did not wish to answer (n (%))	5 (4.8)	11 (9.6)
Gravidity ^†^			
	1 (n (%))	46 (44.2)	42 (36.5)
	2 (n (%))	32 (30.8)	38 (33.0)
	3 (n (%))	14 (13.5)	23 (20.0)
	³ 4 (n (%))	12 (11.5)	12 (10.4)
Parity ^*, †^			
	0 (n (%))	66 (63.5)	NA
	1 (n (%))	26 (25.0)	60 (52.6)
	2 (n (%))	10 (9.6)	42 (36.8)
	³ 3 (n (%))	2 (1.9)	12 (10.4)

Median rank and dollars allocated to each of the LEA outcomes are presented in Table 3 and Table 4. ‘Achieving desired pain relief’ was ranked as the most important outcome by both cohorts. The second-ranked preference was ‘overall satisfaction with the pain management.’ These two outcomes were also assigned the highest relative value by both cohorts. Avoiding various side effects - anxiety, leg weakness, and itching were less important preferences by both priority-ranking and relative value scale allocation in both the antenatal and postpartum cohorts (Table 3 and Table 4).

**Table 3:** Priority ranking and relative value scoring in the antenatal cohort for the labor epidural analgesia (LEA) outcomes evaluated Data are presented as median (x (25%) to y (75%)). Rank: 1 to 10 from the highest priority (1) to the least (10). Relative value: dollar value patients would pay out of $100 to achieve an outcome.

	Priority Ranking (N = 105)	Relative Value Score (N = 105)
Achieving desired pain relief	1 (1 - 3)	30 (18 - 50)
Overall satisfaction with the pain management	4 (2 - 5)	10 (0 - 20)
Experiencing a short duration of labor	5 (3 - 7)	5 (0 - 20)
Experiencing a short time to achieve pain relief	5 (3 - 7)	5 (0 - 10)
Avoiding complications such as low blood pressure	6 (3 - 7)	3 (0 - 10)
Avoiding nausea and/or vomiting as a side effect	6 (4 - 8)	1 (0 - 10)
Receiving the smallest effective dose of pain medication	6 (3 - 9)	3 (0 - 10)
Avoiding anxiety related to labor pain	7 (4 - 9)	1 (0 - 10)
Avoiding leg weakness as a side effect	7 (6 - 9)	0 (0 - 5)
Avoiding itching as a side effect	9 (8 - 10)	0 (0 - 2)

**Table 4:** Priority ranking and relative value scoring in the postpartum for the labor epidural analgesia (LEA) outcomes evaluated Data are presented as median (x (25%) to y (75%)). Rank: 1 to 10 from the highest priority (1) to the least (10). Relative value: dollar value patients would pay out of $100 to achieve an outcome.

	Priority Ranking (N = 114)	Relative Value Score (N = 113)
Achieving desired pain relief	1 (1 - 1)	50 (25 - 60)
Overall satisfaction with the pain management	3 (2 - 4)	10 (0 - 20)
Experiencing a short duration of labor	5 (4 - 7)	5 (0 - 10)
Experiencing a short time to achieve pain relief	3 (2 - 5)	10 (0 - 16)
Avoiding complications such as low blood pressure	6 (5 - 8)	3 (0 - 5)
Avoiding nausea and/or vomiting as a side effect	6 (5 - 8)	2 (0 - 5)
Receiving the smallest effective dose of pain medication	7 (4 - 8)	0 (0 - 5)
Avoiding anxiety related to labor pain	6 (4 - 8)	2 (0 - 8)
Avoiding leg weakness as a side effect	8 (6 - 9)	1 (0 - 5)
Avoiding itching as a side effect	9 (7 - 10)	0 (0 - 5)

There were several differences between the antenatal and postpartum cohorts when comparing the outcome data. The highest-ranked outcome of ‘achieving desired pain relief’ was valued more by the postpartum cohort who gave it a median value of $50 ($25-$60) while the antenatal cohort gave it a median value of $30 ($18-$50). The postpartum cohort ranked ‘experiencing a short time to achieve pain relief’ as more important compared to the antenatal cohort (Median 5 (3-7) vs 3 (2-5)). In exchange, ‘avoiding complications such as low blood pressure’ was ranked lower in the postpartum cohort compared to the antenatal cohort (Median 6 (3-7) vs 6 (5-8)).

Data on expected compared to experienced levels of satisfaction, pre-LEA pain, and post-LEA pain are presented in Table 5 and Table 6. The postpartum cohort reported greater LEA satisfaction than the antenatal cohort indicated they were expecting; a median score of 9 (8-10) out of 10 compared to an 8 (7-10). While the level of expected or experienced pre-LEA pain was different between the two cohorts, the postpartum cohort experienced less post-LEA pain than the antenatal cohort expected. The postpartum cohort gave their post-LEA pain a median score of 2 (1-4) out of 10 compared to the expected score of 4 (2-6) given by the antenatal cohort.

**Table 5:** Expected vs. experienced levels of satisfaction, pre-labor epidural analgesia (LEA) pain, and post-LEA pain in the antenatal cohort LEA = labor epidural analgesia. Data are presented as median (x (25%) to y (75%)) on a scale from 1 to 10 where 1 = lowest satisfaction/pain and 10 = highest satisfaction/pain. * N = 103 for the antenatal cohort. † N = 113 for postpartum cohort.

	Antenatal (N = 104)
Level of satisfaction expected/experienced with LEA	8 (7 - 10)
Level of pain during labor expected/experienced prior to LEA ^*^	10 (8 - 10)
Level of pain during labor expected/experienced after LEA ^*^	4 (2 - 6)

**Table 6:** Expected vs. experienced levels of satisfaction, pre-labor epidural analgesia (LEA) pain, and post-LEA pain in the postpartum cohort LEA = labor epidural analgesia. Data are presented as median (x (25%) to y (75%)) on a scale from 1 to 10 where 1 = lowest satisfaction/pain and 10 = highest satisfaction/pain. * N = 103 for the antenatal cohort. † N = 113 for postpartum cohort.

	Postpartum (N = 115)
Level of satisfaction expected/experienced with LEA	9 (8 - 10)
Level of pain during labor expected/experienced prior to LEA	9 (8 - 10)
Level of pain during labor expected/experienced after LEA ^†^	2 (1 - 4)

## Discussion

After investigating patient preferences for outcomes traditionally associated with LEA, we discovered that the most important outcome for patients was ‘achieving the desired level of pain relief.’ While this finding may seem unsurprising, as it is the intended outcome of the LEA, we believe it is important to confirm this preference from a patient perspective and to realize that concerns for potential side effects are secondary.

Previous research on this topic is limited; however, the data that exist are consistent with our findings. Carvalho et al. evaluated the preferences of patients undergoing scheduled cesarean deliveries and found that pain was the greatest patient concern [7]. Similarly, these patients assigned lesser value to side effects of their spinal anesthesia [7]. A study assessing the importance laboring patients apply to pain intensity versus the duration of pain found patients preferred lower pain intensity at the cost of longer pain duration [10]. Preferencing pain intensity over duration is reinforced in our postpartum cohort. Our participants did not alter their ranking of “shorter duration of labor,” but significantly increased their prioritization of pain management by increasing the preference for a shorter duration to effective pain relief. This was also evaluated by Favilli et al. who assessed patient preference regarding pain and duration of labor [11]. Patients who delivered with epidural analgesia significantly preferred less pain over a longer duration of labor compared to their pre-labor counterparts [11].

Of note, our results differ from Sutton et al. who queried Google Trends data for the United States to evaluate the most common searches for epidural and birth-related information and determine temporal trends [12]. ﻿Searches for epidural side effects, risks, and pain on insertion were among the most common [12] while avoiding side effects was of least concern for the participants in our study.

As previously stated, our objective with this research was to determine patient preferences for labor epidural outcomes, however, we limited our ability to truly assess patient preferences by restricting the outcomes to those used in previous research. Future research should engage patients as members of the research team to ensure patient-centered metrics are used or employ a qualitative methodology to further characterize their experiences. Our study was also limited by time, and we were unable to follow the same cohort of patients through their pregnancy and assess their preferences both antenatally and postpartum; this should be considered in future research.

With our study, we aimed to align our methodology with that of a previous Carvalho et al. study, which evaluated the preferences of patients undergoing scheduled cesarean deliveries [7]. However, in doing so, we used a different approach for the relative value scale questions than Engoren et al. did [9]. While we assumed the dollar amount assigned to be a relative value score or a willingness to pay, Engoren et al. asked "How much would you pay to completely eliminate each of those problems?” [9]. This difference in methodology will affect the ability to compare the results of various studies.

A further potential limitation of this research is the study population, which may affect the generalizability of the results. The majority of participants were Caucasian, married or living with their partner, highly educated, and reported a household income greater than $88,000 per year. While this is consistent with the patient population at our institution, it does not necessarily represent the average obstetrical patient at other centers across North America. However, attitudes toward LEA appear to be unrelated to sociodemographic factors [4]. The development of patient-centered outcomes for labor analgesia needs to ensure a diverse and inclusive methodology to maximize generalizability.

Another potential limitation is the censoring of incorrectly completed questionnaires. A number of participants had difficulty completing the questionnaire, resulting in 77 incorrectly completed questionnaires. This mostly happened in the priority ranking and relative value scale sections. Common mistakes included repeating and/or missing ranks, spending too much or too little money, and inverting the rank or money system whereby more money was allocated to higher-ranked outcomes. Excluding these questionnaires was the a priori plan and may have resulted in responder biases. Additional questionnaire development with an increased focus on the instructions provided and the readability of the questionnaire by more patients may have improved this. Furthermore, an additional question with the purpose of identifying reading and comprehension might have better objectified this process. However, the majority of participants completed the questionnaire, and we obtained a sufficient sample size in both cohorts to achieve adequate power.

Furthermore, patients with a positive LEA experience may have been more likely to participate. While we collected some data on obstetrical history and childbirth experience, we did not explore the relationship between these factors and outcome preferences. We also did not collect data on how many participants experienced the LEA outcomes used in the study, which may have affected the results. Investigating whether experiencing the LEA outcomes, parity, previous LEA use, or a difficult birth experience affect preferences would help further improve and personalize the LEA experience.

## Conclusions

In conclusion, our study found that achieving the desired level of pain relief was the greatest LEA-related outcome preference for both antenatal and postpartum patients. Side effects, such as leg weakness and pruritus, were less important preferences relative to pain-related outcomes. Our results show that it is the success of the intended outcome, as well as timely provision and overall satisfaction, which should be the primary driver when providing LEA. The results of this study will inform both future LEA research, as well as physician and other healthcare providers’ discussions with patients.
